# Supplementary material for: Influence of Natural Mordenite Activation Mode on Its Efficiency as Support of Nickel Catalysts for Biodiesel Upgrading to Renewable Diesel
Source: Nanomaterials (Basel). 2023 May 10;13(10):1603. doi: 10.3390/nano13101603 (PMC10221656; doi:10.3390/nano13101603)
Supplement: Supplementary file 1 [file nanomaterials-13-01603-s001.zip › nanomaterials-2346757-supplementary.pdf]

Influence of natural mordenite activation mode on its efficiency as support of nickel catalysts for biodiesel upgrading to renewable diesel

Konstantina Fani[1], Sotiris Lycourghiotis[1], Kyriakos Bourikas[1], Eleana Kordouli[1,2]*

[1] School of Science and Technology, Hellenic Open University, Parodos Aristotelous 18, 26335 Patras, Greece

[2] Department of Chemistry, University of Patras, 26504 Patras, Greece

*Corresponding Author: ekordouli@upatras.gr

Preparation of nickel catalysts supported on activated mordenite

The catalysts were prepared following the deposition-precipitation method. More precisely, a weighted amount of $Ni(NO_3)_2 \times 6H_2O$ was dissolved in 50 mL distilled water in a spherical flask (250 mL) and then a weighted amount of activated mordenite was added. 50 mL of $CO(NH_2)_2$ aqueous solution was added. The final concentration of urea molecules was triple of that of the NO_3^- ions coming from the nickel salt. A reflux condenser and a magnetic stirrer were installed, and the spherical flask was heated in an oil bath at 100 °C for 10 h. After cooling down to ambient temperature the suspension was vacuum filtered.

The solids obtained in all cases were dried at 110 °C overnight, heated under argon flow (30 mL/min) at a heat rate of 10 °C/min to 500 °C, and then they were reduced at 500 °C for 2.5 h under H_2 flow (30 mL/min). The obtained catalysts contain 30 wt. % Ni and are symbolized as $NiMO_x$, where x represents the activation mode of natural mordenite (A or AB).

Catalysts' characterization

X-Ray Fluorescence spectroscopy (XRF) was used to measure the supports and catalysts composition using a benchtop Total-reflection X-Ray Fluorescence spectrometer (S2 PICOFOX™, Bruker Nano GmbH, Germany).

Nitrogen adsorption - desorption isotherms were recorded in a Micromeritics apparatus (Tristar 3000 porosimeter). Specific surface area (S_{BET}) calculation was based on BET equation using adsorption data recorded at relative N_2 pressure in the range $0.03 < P/P_o <$

0.5. The microporous specific surface area (S_{micro}) was calculated by the t-plot method. Pore size distribution was determined using the BJH method and the N_2 desorption curve.

X-ray powder diffraction (XRD) patterns were recorded in the range of 10° ≤ 2θ ≤ 80° by a Bruker D8 Advance diffractometer equipped with nickel - filtered CuKα (0.15418 nm) radiation source working at 40 kV and 40 mA. The step size and the time per step were respectively fixed at 0.02° and 0.5s. The mean crystallite size was estimated using Scherrer's equation.

Scanning Electron Microscopy - Energy Dispersive Spectrometry (SEM-EDS) was used to obtain microphotographs of the catalysts and to confirm the percentage amount of nickel in the catalysts. Scanning Electron Microscope (SEMJEOL JSM6300) equipped with an Energy Dispersive Spectrometry accessory has been used working with 20 kV accelerating voltage and 10 nA beam current. Microanalysis was performed on gold coated samples. The sample powders were mounted directly on the sample holder.

Transmittance Electron Microscopy (TEM) was used for recording TEM images of the catalysts and determine the mean nickel particle size in a JEOL JEM-2100 system operated at 200 kV (resolution: point 0.23 nm, lattice 0.14 nm) equipped with an Erlangshen CCD Camera (Gatan, Model 782 ES500W). The specimens were prepared by dispersion in water and spread onto a carbon-coated copper grid (200 meshes).

NH_3-TPD experiments were performed in a laboratory-constructed equipment. In a typical experiment, 100 mg of the pre-reduced catalyst were placed in a quartz micro-reactor and helium was fed (flow rate 30 mL min^{-1}) for 30 min in order to remove any adsorbed species from the catalyst surface. Then, a stream of NH_3 was fed in the micro-reactor for 30 min at room temperature and then it was switched to He to remove the physically adsorbed ammonia. The temperature was then increased linearly (10 °C/min) up to 600 °C. The amount of the desorbed ammonia was determined by a thermal conductivity detector (TCD).

H_2-TPR Precursor samples (after Ar treatment at 400 °C and before reduction) were characterized by H_2-TPR experiments performed in the same laboratory-constructed equipment. 0.04 g of the sample was placed in the quartz reactor and the reducing gas mixture (H_2/Ar: 5/95 v/v) was passed through it for 2 h, with a flow rate of 40 mL min^{-1}

at room temperature. Then the temperature was increased to 1000 °C with a constant rate of 10 °C min^{-1}. Reduction leads to a decrease of the hydrogen concentration of the gas mixture, which was detected by the TCD. The reducing gas mixture was dried in a cold trap (−95 °C) before reaching the TCD.

Combustion elemental analysis The total carbon (TC) content of a sample was determined by a High Temperature - Dry Combustion Technique, using a Shimadzu TOC-L_{CSH} main unit and a SSM-5000A sample combustion unit (Shimadzu, Kyoto, Japan) with a NDIR detector. Initially, the sample is weighted into a ceramic boat. It is inserted into the TC furnace of the instrument and combusted at 900ºC in a stream of oxygen. Cobalt / Platinum mixed catalyst is used to ensure and accelerate combustion. The generated gases are passed through a drain vessel and a halogen scrubber, which remove water-vapor and halogenated compounds, respectively. The carbon dioxide is measured to the NDIR detector and finally trapped/removed by CO_2 absorber (soda).

Catalysts' evaluation

The catalysts were evaluated for the transformation of biodiesel to green diesel under solvent free conditions, in a high-pressure semi-batch reactor (300 mL, Autoclave Engineers). Biodiesel volume to catalyst mass ratio was equal to 100 mL/g and the speed of stirring was 1000 rpm. The reactor was heated with a temperature rate of 10 ˚C/min at the reaction temperature (310, 330 and 350˚C, accordingly) under Ar flow (100 mL/min) to purge the dead volume from the ambient air. When the desired reaction temperature was achieved, the Ar stream was changed to H_2 with the same flow rate, controlled by a mass flow controller (Brooks 58505S). The hydrogen pressure was maintained constant during the 9 h experiment at 40 bar.

A gas chromatograph (Shimadzu GC-2010 Plus) equipped with a flame ionization detector (FID) and a ZB-5HT INFERNO, ZEBRON (l: 30m, d: 0.32mm tf: 0.10μm) column, working in a split mode (split ratio: 40) was used for the liquid product analysis. The temperature pattern used in the oven of the chromatograph, from 50 ˚C to 380 ˚C, has been described elsewhere [Ref. 28 in the article]. The injector temperature was set at 350 ˚C and that of the detector at 390 ˚C. Heptane (Sigma Aldrich, ≥99% n-heptane basis) was used for dilution (1:20) of the liquid samples withdrawn from the reactor and as external standard for quantification of the results. 1 μL of the diluted sample was injected into the GC. The product identification was confirmed with gas

chromatography - mass spectrometry (Shimadzu GCMS-QP2010 Ultra). Gas-phase analysis was performed online by using a six-port sampling valve and a Shimadzu GC-8A chromatograph equipped with a thermal conductivity detector and an appropriate column (SUPELCO, 110/120 Carbosieve S II, 10F, 1/8 in).

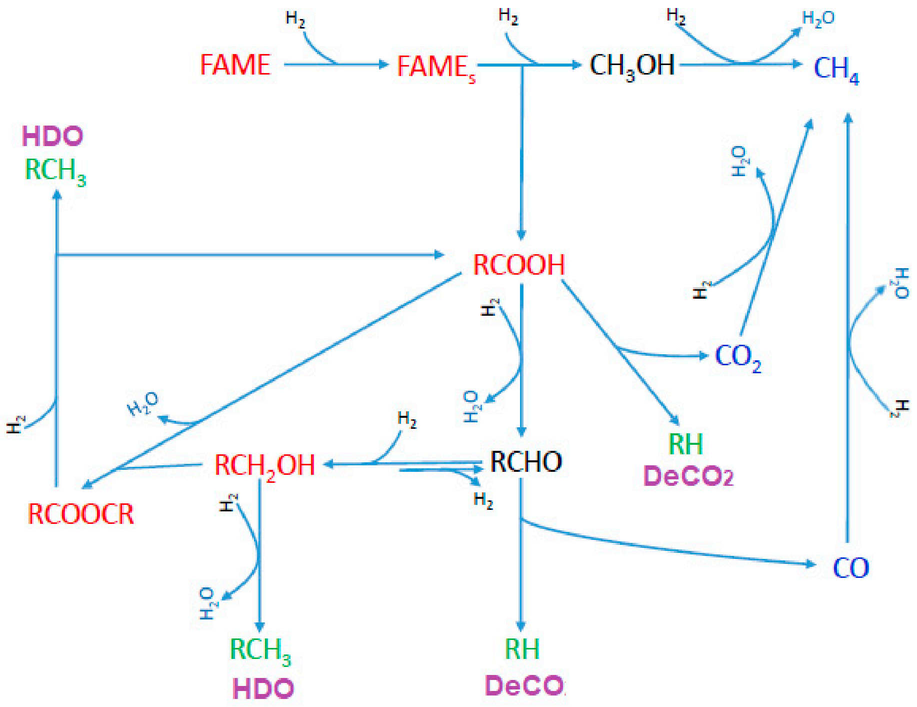

Scheme S1. Reaction pathways upon biodiesel transformation to renewable diesel over Ni supported catalysts.

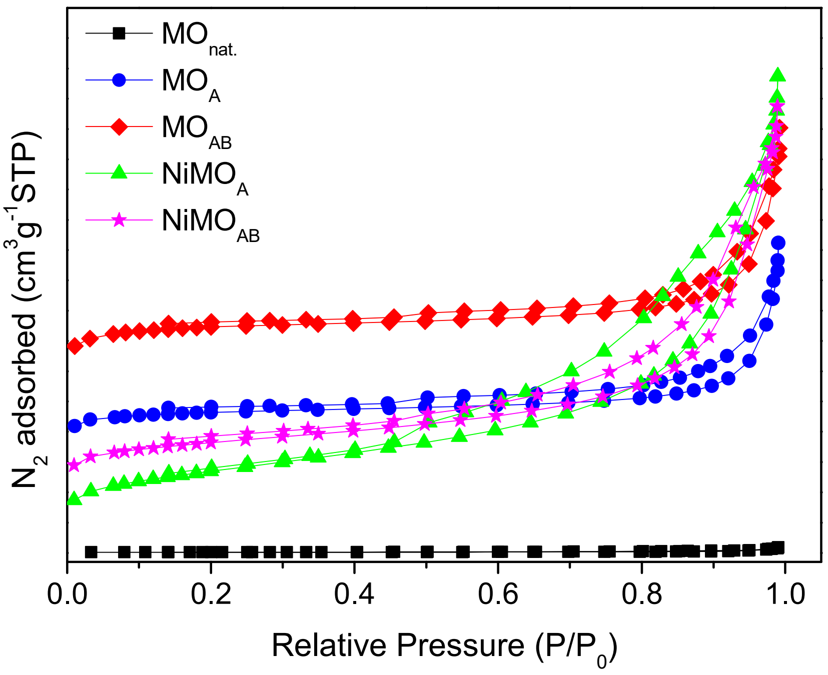

Figure S1. N_2 adsorption – desorption isotherms of natural mordenite, acid activated mordenite, acid – base activated mordenite and the corresponding nickel/mordenite catalysts.

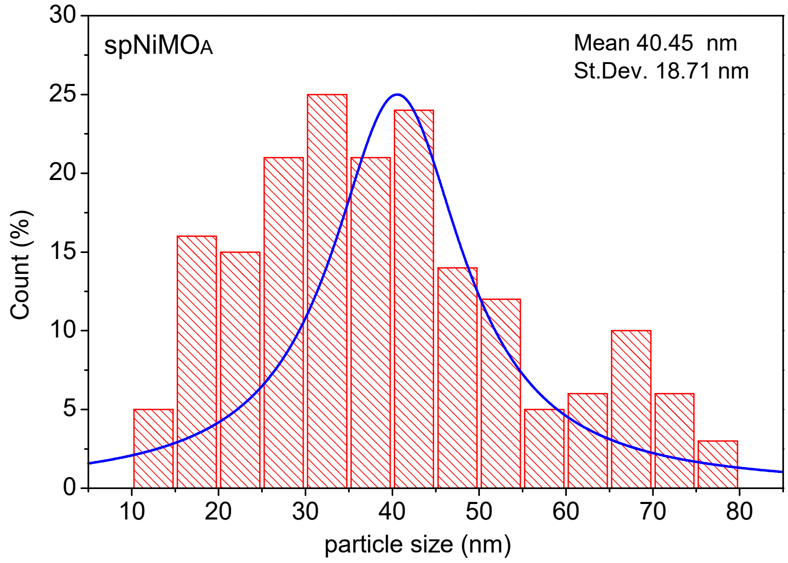

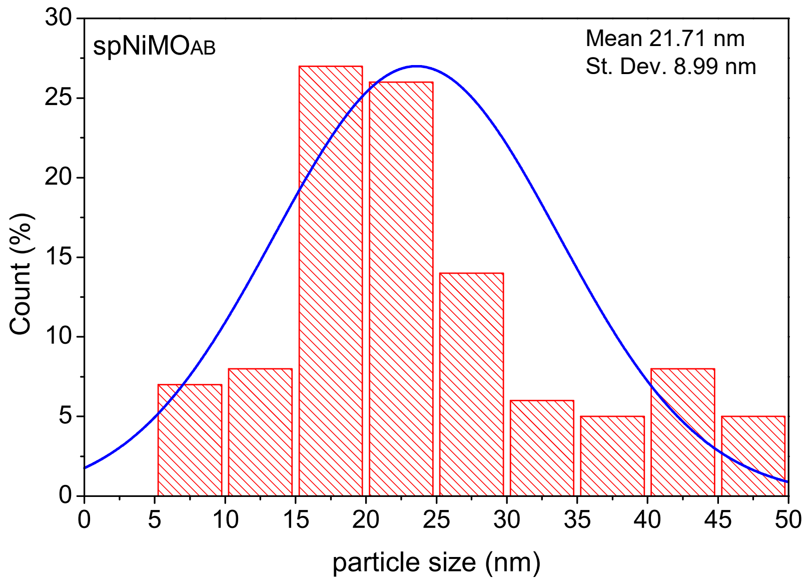

Figure S2. Nickel particle size distributions for the spNiMO$_A$ and spNiMO$_{AB}$ catalysts, after 9h of reaction at 310 °C.

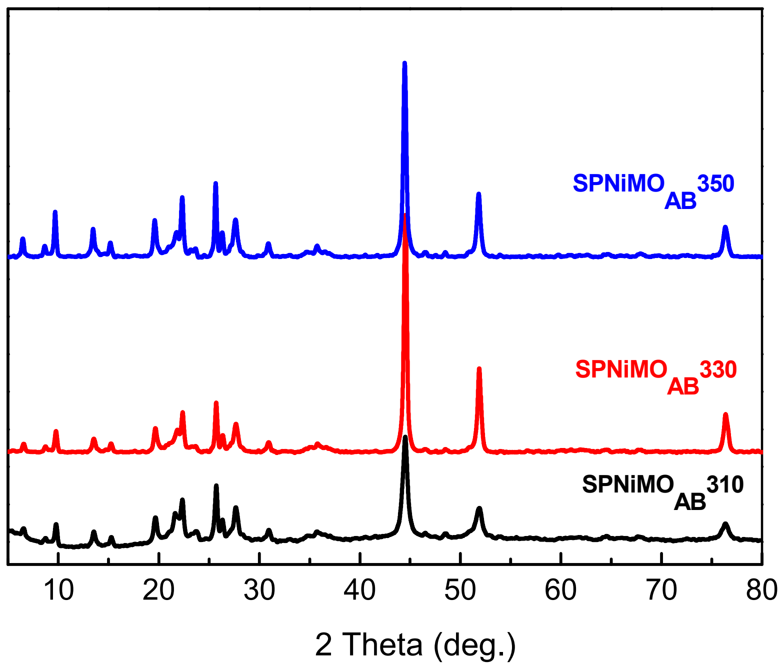

Figure S3. XRD patterns of the spent NiMO$_{AB}$ catalyst, after reaction at various temperatures.

Table S1 Values of reaction parameters determined over the $NiMO_{AB}$ catalyst, at various reaction temperatures.

Reaction temperature (ºC)	Reaction time (h)	Conversion (%)	Hydrocarbons (wt. %)	Acids (wt. %)	Esters (wt. %)	C15 (wt. %)	C16 (wt. %)	C17 (wt. %)	C18 (wt. %)
310	2	81.4	17.4	22.8	41.2	1.35	0.4	15.1	0.55
	4	88.3	24.8	19.6	43.9	1.5	0.5	20.7	2.1
	6	92	37.5	14.1	40.4	2.4	0.75	30.1	4.25
	7	94.7	42.3	12.6	39.8	2.6	0.8	33.5	5.4
	8	95.2	46.8	9.4	39	2.9	0.87	37	6.03
	9	96.5	51.9	8.3	36.3	3.29	0.98	40.54	7.09
330	2	83	21.8	34.5	26.7	1.4	0.8	15.6	3.7
	4	89.5	30.6	29.8	29.1	1.8	1	21.4	6.4
	6	95.1	46.3	21.2	27.6	2.5	1.5	31.1	10.9
	7	96.6	52.2	17.9	26.5	2.8	1.7	34.6	12.8
	8	97.2	58.1	14.3	24.8	3	1.9	38.2	14.6
	9	97.6	64.1	12.6	20.9	3.3	2.1	41.9	16.8
350	2	83	33.1	35.6	14.3	2.1	1.7	21.8	7.5
	4	95.3	56.3	29.1	9.9	4.1	2.5	36.8	12.9
	6	99	76.3	18.2	4.5	5	3.2	50.1	18
	7	99.3	84.1	12.1	3.1	5.3	3.5	55.6	19.7
	8	99.3	89.6	7	2.7	5.6	3.6	59.4	21
	9	99.4	93.8	3.5	2.1	5.7	3.7	62.5	21.9
